# Probiotic Properties of *Bifidobacterium longum* KABP042 and *Pediococcus pentosaceus* KABP041 Show Potential to Counteract Functional Gastrointestinal Disorders in an Observational Pilot Trial in Infants

**DOI:** 10.3389/fmicb.2021.741391

**Published:** 2022-01-12

**Authors:** Erola Astó, Pol Huedo, Tatiana Altadill, Meritxell Aguiló García, Maura Sticco, Marta Perez, Jordi Espadaler-Mazo

**Affiliations:** ^1^R&D Department, AB-Biotics S.A. (Part of Kaneka Corporation), Barcelona, Spain; ^2^Basic Sciences Department, Universitat Internacional de Catalunya, Barcelona, Spain; ^3^Pediatric Primary Care Local Health Authority, ASL Caserta, Caserta, Italy

**Keywords:** probiotics, antimicrobial activity, intestinal epithelium, tight junction proteins, FGID, constipation, infant colic, pediatric

## Abstract

Functional gastrointestinal disorders (FGIDs) are a common concern during the first year of life. Recognized as gut-brain axis disorders by Rome IV criteria, FGIDs etiology is linked to altered gut-brain interaction, intestinal physiology, and microbiota. In this regard, probiotics have emerged as a promising therapy for infant FGIDs. In this study, we have investigated the probiotic potential of the strains *Bifidobacterium longum* KABP042 and *Pediococcus pentosaceus* KABP041—isolated from healthy children’s feces—in the treatment of FGIDs. To this scope, genome sequences of both strains were obtained and subjected to *in silico* analyses. No virulence factors were detected for any strain and only the non-transferable *erm(49)* gene, which confers resistance to erythromycin and clindamycin, was identified in the genome of *B. longum* KABP042. Safety of both strains was confirmed by acute oral toxicity in rats. *In vitro* characterization revealed that the strains tolerate gastric and bile challenges and display a great adhesion capacity to human intestinal cells. The two strains mediate adhesion by different mechanisms and, when combined, synergically induce the expression of Caco-2 tight junction proteins. Moreover, growth inhibition experiments demonstrated the ability of the two strains alone and in combination to antagonize diverse Gram-negative and Gram-positive bacterial pathogens during sessile and planktonic growth. Pathogens’ inhibition was mostly mediated by the production of organic acids, but neutralization experiments strongly suggested the presence of additional antimicrobial compounds in probiotic culture supernatants such as the bacteriocin Lantibiotic B, whose gene was detected in the genome of *B. longum* KABP042. Finally, an exploratory, observational, pilot study involving 36 infants diagnosed with at least one FGID (infant colic and/or functional constipation) showed the probiotic formula was well tolerated and FGID severity was significantly reduced after 14 days of treatment with the 2 strains. Overall, this work provides evidence of the probiotic and synergic properties of strains *B. longum* KABP042 and *P. pentosaceus* KABP041, and of their potential to treat pediatric FGIDs.

**Clinical Trial Registration:** [www.ClinicalTrials.gov], [identifier NCT04944628].

## Introduction

Infants commonly suffer from gastrointestinal symptoms that do not have an organic explanation and are thus diagnosed as having a functional gastrointestinal disorder (FGID). One in two infants develop FGIDs during the first 6 months of life and up to 75% of these children suffer two or more at the same time (Vandenplas et al., 2015). FGIDs reduce quality of life of pediatric patients (Robin et al., 2018), cause distress to caregivers impairing family function and lead to repetitive visits to pediatricians impacting healthcare costs (Vandenplas et al., 2015). Infant colic and functional constipation are among the most prevalent pediatric FGIDs, with an aggregated prevalence of 35% in infants younger than 12 months (Vandenplas et al., 2015). Infant colic, also called excessive crying syndrome, consists of prolonged, intense, and inconsolable crying or fussiness presented by otherwise healthy infants up to 5 months of life. Although benign and self-resolved condition, colic strongly impacts the quality of life of the family by triggering parental anxiety and increasing the risk of maternal depression, shaken baby syndrome and premature breastfeeding cessation (Sung, 2018). Functional constipation involves infrequent and/or painful defecation, fecal incontinence, and abdominal pain, causing physical suffering to infants and emotional suffering to infants and parents (Levy et al., 2017).

With an unclear underlying pathophysiology, FGIDs have been described by Rome IV committee criteria as gut-brain axis disorders caused by complex interactions between gut physiology and physiological factors that ultimately alter intestinal motility, sensitivity and barrier function (Drossman, 2016). Gut microbiome has emerged as a central player in the gut-brain axis and thus in the FGIDs etiology. Microbiota of FGID patients differs from healthy controls and aberrant neonatal gut colonization is suggested to precede development of some FGIDs (Pärtty et al., 2018; Mukhtar et al., 2019). The gut of colicky babies has reduced diversity, higher levels of Gram-negative bacteria and lower numbers of *Bifidobacteria* and *Lactobacillus* (de Weerth et al., 2013; Savino et al., 2017; Rhoads et al., 2018). Some data suggest gut microbiota disturbances in infants with functional constipation with different results among studies (Vriesman et al., 2020).

In this context, probiotics have been proposed as a potential therapy for FGIDs and as an alternative to pharmacologic treatments, whose usefulness is scarce and debatable (Benninga et al., 2016; Pärtty et al., 2018). Nevertheless, the efficacy of different strains is limited and controversial due to the heterogeneity of studies and poorly known mechanisms of action, urging the need of studies with well-characterized strains (Sung, 2018).

The aim of this study was to investigate the *in silico, in vitro*, and *in vivo* potential of *Bifidobacterium longum* subsp. *longum* KABP042 and *Pediococcus pentosaceus* KABP041 strains, isolated from the feces of healthy children, as probiotics. Safety properties, resistance to gastrointestinal challenges, adherence, gene expression modulation of intestinal epithelium, and antimicrobial activity against pathogens were investigated in depth. Furthermore, an exploratory, observational, pilot trial was set to evaluate the safety, tolerability and potential efficacy of a probiotic formula containing *B. longum* KABP042 and *P. pentosaceus* KABP041 in infants with FGIDs.

## Materials and Methods

### Bacterial Strains and Growth Culture Conditions

*B. longum* subsp. *longum* KABP042 (CECT 7894) was originally isolated from feces of a healthy breast-fed baby and *P. pentosaceus* KABP041 (CECT 8330) from feces of a healthy child. Both strains belonged to our own collection (Cuñé Castellana et al., 2015). *Lacticaseibacillus rhamnosus* GG (ATCC 53103), *B. longum* subsp *longum* ATCC 15707, *Lacticaseibacillus paracasei* ATCC 334, *Streptococcus pyogenes* CECT 191, and *Bacillus cereus* CECT 5144 were obtained from the American Type Culture Collection (ATCC; Manassas, VA) or the Spanish Type Culture Collection (CECT; Valencia Spain) and used as control strains as indicated. Enteropathogenic *Escherichia coli* CECT 729 (EPEC), *Klebsiella pneumoniae* DSM 11678, *Salmonella enterica* subsp. *enterica* serovar *typhimurium* LT2 (ATCC 700720), *Staphylococcus epidermidis* CIP 81.55T, *Staphylococcus aureus* CIP 107860 and *Enterococcus faecalis* ATCC 29212 were obtained from the ATCC, CECT, German Collection of Microorganisms and Cell Cultures (DSM; Braunschweig, Germany) or Institute Pasteur Collection (CIP; Paris, France) and used as indicator strains in the antimicrobial activity assays.

*P. pentosaceus* and lactobacilli strains were routinely grown in MRS (De Man, Rogosa, and Sharpe) broth or agar (Scharlab, Barcelona, Spain) for 18–48 h at 37°C in chambers with anaerobic sachets (Anaerogen, Thermo Scientific, Barcelona, Spain). Bifidobacteria strains were grown in the same conditions except that MRS was supplemented with 0.1% (w/v) cysteine-HCl (Scharlab) (MRScys). *S. pyogenes*, enterobacteria, staphylococci and *E. faecalis* strains were cultured in tryptic soy broth (TSB) or agar (TSA) (Scharlab) for 18–24 h aerobically at 37°C as indicated. *B. cereus* CECT 5144 was grown in similar conditions on nutrient gelatin agar (*per* L: peptone 4 g, yeast extract 1 g, gelatin 40 g and agar 15 g).

### Antibiotics Susceptibility

The antimicrobial susceptibility profile of probiotic strains to a panel of 8 antibiotics required by European Food Safety Authority (EFSA, 2018) was investigated (Table 1). Minimum inhibitory concentrations (MIC) were determined by broth-microdilution method following the standard ISO 10932:2010 IDF 223:210 (2010). *B. longum* ATCC 15707 and *L. paracasei* ATCC 334 were used as control strains. Susceptibility of tested strains was determined by comparing MIC values with cut-off values provided by EFSA in their latest Scientific Opinion (EFSA, 2018).

**TABLE 1:** MIC values (μg/mL) of antibiotics tested against studied strains and susceptibility or resistance based on comparison with EFSA cut-off values.

Antibiotic	*P. pentosaceus* KABP041	*B. longum* KABP042
Ampicillin	4 (S)	0.25 (S)
Gentamicin	8 (S)	32 (S)
Kanamycin	128 (R)	nr
Streptomycin	128 (R)	128 (S)
Erythromycin	0.25 (S)	> 8 (R)
Clindamycin	0.063 (S)	> 16 (R)
Tetracycline	16 (R)	0.5 (S)
Chloramphenicol	4 (S)	2 (S)
Vancomycin	nr	0.5 (S)

*S, susceptible; R, resistant, nr, not required.*

### Quantification of Biogenic Amines Biosynthesis

The ability to produce 4 biogenic amines through 5 biosynthetic routes -putrescine from agmatine and from ornithine, histamine from histidine, cadaverine from lysine and tyramine from tyrosine- was studied. Strains were cultured in MRS or MRScys supplemented with 1 mM of the corresponding precursor. Overnight culture supernatants were recovered by centrifugation. Samples were derivatized with diethyl ethoxymethylenemalonate and filtered through 0.2 μM filters and biogenic amines were quantified by UHPLC as described by Redruello et al. (2013) at IPLA-CSIC (Villaviciosa, Spain). Putrescine, histamine, cadaverine and tyramine (Sigma-Aldrich, Madrid, Spain) were used as standards.

### Hemolytic, Gelatinase, and General Enzymatic Activities

Hemolytic activity was determined by streaking the strains on Columbia blood agar plates containing 5% defibrinated sheep blood (Scharlab). After 48 h of incubation at 37°C the plates were observed for hemolytic reaction (Kondrotiene et al., 2020). *S. pyogenes* CECT 191 was used as positive control. Gelatinase activity was tested by spotting fresh cultures on nutrient gelatin agar plates. Strains were cultured at 37°C for 5 days. Plates were treated with acidic mercuric chloride solution for 10 min to detect opaque halos indicating digestion of gelatin (dela Cruz and Torres, 2012). *B. cereus* CECT 5144 was used as positive control. To test additional enzymatic activities, API^®^ ZYM (bioMérieux, Madrid, Spain) experiments were performed by triplicate following manufacturer instructions.

### Resistance to Gastrointestinal Conditions

Gastric stress and bile salt tolerance assays were performed as described elsewhere (Kondrotiene et al., 2020) with slight modifications. To study the gastric stress resistance, overnight cultures were used to inoculate (1%) simulated gastric solutions (*per* L: NaCl 7.3 g, KCl 0.52 g, NaHCO_3_ 3.78 g and pepsin 3 g) at pH 2.3 and pH 3 adjusted with 1N HCl. Strains were incubated at 37°C for 30 min in condition at pH 2.3 and for 90 min in condition at pH 3. To determine bile salt tolerance, MRS or MRScys broth containing 0.3% (w/v) bile salts (Sigma-Aldrich) were inoculated at 1% from overnight cultures and incubated for 180 min at 37°C. Routine media inoculated at 1% were used as controls of active cultures. Proliferative bacteria were counted before and after incubation times by serial dilution and counting method on MRS or MRScys agar plates. The plates were incubated for 48 h. Commercial probiotic strain *L. rhamnosus* GG was used as reference. Results were expressed as mean and standard deviation of log CFU/mL. Determinations were performed in independent triplicates.

### Adherence to Intestinal Epithelial Cells

The ability of probiotic strains to adhere to the intestinal epithelium was studied *in vitro* using Caco-2 intestinal epithelial cells. Routinely, cells were grown in DMEM High Glucose with 10% decomplemented fetal calf serum (FCS), 40 μg/mL gentamycin and 50 μg/mL L-glutamine (Euroclone, Milan, Italy) at 37°C in a 5% CO_2_ atmosphere. Caco-2 cells were seeded in 24-well plates and cultivated until confluence. Caco-2 cells were washed with HBSS (hank’s balanced salt solution, Sigma-Aldrich) and incubated for 1 h with 875 μL of DMEM + 1% FCS under the same conditions. Bacterial cells from overnight cultures were recovered by centrifugation, washed twice with sterile distilled water, and suspended in sterile DMEM + 1% FCS at 1.5 × 10^7^ CFU/mL. Bacterial suspensions were added to Caco-2 monolayers (MOI 1:5 cells to probiotic) and to wells without Caco-2 cells as controls. After 1 h of incubation at 37°C with 5% CO_2_, the medium was removed, Caco-2 cells were washed 3 times with HBSS for 5 min and detached by adding 100 μL of trypsin for 5 min at 37°C, and finally recovered with 900 μL of MRD (Maximum Recovery Diluent, BD Difco). Bacterial suspensions were enumerated by plating serial dilutions on MRS or MRScys. Bacteria in the medium of the control wells were also quantified and bacterial adhesion capacity was calculated as the percentage of log CFU/ml of adhered bacteria relative to log CFU/ml in the controls. The experiments were performed in two independent replicates. *L. paracasei* ATCC 344 and *B. longum* ATCC 15707 were used as quality controls with a known adhesion percentage of 64–71 and 47–55, respectively (Sagheddu et al., 2020).

### Analysis of Gene Expression of Caco-2 Cells

To study the effect of probiotics on expression of Caco-2 genes, differentiated Caco-2 cells were washed with HBSS and left for 1 h in DMEM. Cells were exposed to single and combined bacterial suspensions (prepared by mixing equal volumes of each strain) for 5 h at 37°C in a 5% CO_2_ atmosphere (MOI 1.5:1/cells to probiotic). Caco-2 wells without probiotic addition were used as controls. At the end of the incubation period, total RNA was extracted with Aurum Total RNA Mini Kit (Bio-Rad, Barcelona, Spain) following manufacturer’s instructions and quantified with NanoDrop (Thermo Fisher Scientific). cDNA was obtained from 2 μg of RNA through the IScript Advanced cDNA Syntesis Kit (Bio-Rad). Gene expression was quantified using 100 ng of cDNA by Reverse Transcription Quantitative PCR (RT-qPCR) with SsoAdvanced Universal SYBR Green Supermix (Bio-Rad). RT-qPCR program was set as follows: 50°C for 2 min; 95°C for 7 min; 40 cycles of 95°C for 15 s, 60°C for 1 min and 72°C for 1 min; and 95°C for 10 s. Target genes included zonula ocludens-1 (ZO-1) ocludine (OCLN), claudine (CLDN1) and serotonine trasporter SERT (SLC6A4). β-actin gene was measured as internal control for normalization. Gene-specific primers were obtained from Bio-Rad (PrimePCR SYBR Green Assay) with the following codes: qHsaCID0018062 for ZO-1, qHsaCED0038290 for OCLN, qHsaCID0006097 for CLDN-1, qHsaCID0016255 for SLC6A4 and qHsaCED0036269 for β-actin. Relative mRNA expression was calculated using the 2^ΔΔ*Ct*^ method. RT-qPCR analysis was performed on RNA purified from two independent replicates for each condition.

### Determination of the Antimicrobial Activity

The ability of probiotic strains to inhibit the growth of Gram-positive and Gram-negative pathogens was studied in liquid cultures by microplate growth inhibition assay using supernatants of probiotic mono-cultures and probiotic + pathogen co-cultures, and in agar cultures by overlay assay.

To obtain mono-culture supernatants of probiotic strains, overnight cultures adjusted to an optical density (OD_620 *nm*_) of 0.2 were inoculated (0.1%) into tubes containing 2x concentrated MRS or MRScys and incubated for 16 h at 37°C in anaerobiosis. Then, one volume of 2x TSB medium was added to the probiotic culture and the reconstituted mixture was further incubated for 24 h under the same conditions. Supernatants of pure *P. pentosaceus* and *B. longum* cultures were collected by centrifugation, filtered and an aliquot was neutralized with 5N NaOH.

To investigate whether the antimicrobial activity of probiotic liquid cultures could be induced by the presence of particular pathogens (Maldonado-Barragán et al., 2016), probiotic + pathogen co-cultures were performed. To this end, probiotic and pathogen overnight cultures (OD_620 *nm*_ of 0.2) were inoculated (0.1%) into tubes containing 2x MRS or MRScys (for probiotic strains) or 2x TSB medium (for pathogens) and incubated for 16 h at 37°C in anaerobiosis and aerobiosis, respectively. Pure probiotics and pathogens cultures were then mixed 1:1 and co-cultures were incubated anaerobically for an additional 24 h. Supernatants of mixed probiotic-pathogen cultures were collected and processed as indicated above.

The inhibitory activity of mono-culture and co-culture supernatants against pathogens’ growth was tested in 96-wells plates assays as described by Vijayakumar and Muriana (2015). Overnight cultures of bacterial pathogens were adjusted to an OD_620 *nm*_ of 0.2 in 2x TSB medium and 100 μL were poured into microplate wells. One hundred μL of crude and neutralized mono-culture and co-culture supernatants were added to the wells containing indicator strains. To investigate the potential synergistic effect of *P. pentosaceus* and *B. longum* culture supernatants, 50 μL of each crude or neutralized supernatant (1:1) were added to the wells. As controls, wells containing pathogens cultures were supplemented with 50 μL of 2x MRS and 50 μL of 2x TSB. The OD_620 *nm*_ of the resulting cultures was monitored for 16 h using a plate reader and growth curves were obtained. The antagonistic activity of the different supernatants was quantified by calculating the Area Under the Curve (AUC) of the treated cultures in relation to that of the control wells and expressed as relative growth inhibition in percentage. The experiment was performed at least by duplicate.

Agar overlay method was performed as described previously (Magnusson and Schnürer, 2001) with modifications adapted from Lee et al. (2011). Overnight cultures of *B. longum* KABP042 and *P. pentosaceus* KABP041 were adjusted to an OD_620 *nm*_ of 0.8. Ten μL of each strain and a mixture (1:1) of both were spot-inoculated on MRScys agar plates. After 24 h of incubation, pathogenic strains (10^9^ CFU approximately) were suspended in soft TSA (0.9% agar) with and without buffering with 0.5 M Tris and poured onto spot-inoculated MRScys agar plates. Plates were further incubated for 48 h and the zones of inhibition were measured. The experiment was performed in two independent occasions.

Finally, to verify the proteinaceous nature of the antimicrobial molecules, neutralized mono-culture supernatants were individually treated with proteinase K (Thermo Fisher Scientific) (1 mg/mL) and trypsin (Sigma-Aldrich) (5 mg/mL) at 37°C for 2 h. Enzymes were inactivated by boiling for 3 min. Treated supernatants were tested by microplate growth inhibition assay.

### Quantification of Lactate and Acetate Production

Probiotic strains were grown in MRS or MRScys for 24 h in anaerobic conditions at 37°C. Next day, culture supernatants were filtered, and lactate and acetate concentrations were determined by ion chromatography at Analysis Service Unit facilities of ICTAN-CSIC (Madrid, Spain). L-lactic acid isomer was quantified by enzymatic kit (Biosystems, Barcelona, Spain) according to manufacturer’s instructions. Assays were performed in three different occasions.

### Genome Sequencing and *in silico* Analyses

#### Whole Genome Sequencing

The whole genome sequences (WGS) of *B. longum* KABP042 and *P. pentosaceus* KABP041 were obtained by Illumina HiSeq 2500 using a paired-end library preparation of 2 × 125 bp and considering an insert size of 500 bp. Quality based trimming of raw Illumina data was performed to remove bases having PHRED quality score of<30 and any reads shorter than 20 bp. *De novo* genome assembly of Illumina data was performed using SPAdes version 3.12.0 (Bankevich et al., 2012) using pre-correction, careful and multi k-mer options. Genome sequences were annotated using DFAST (Tanizawa et al., 2018). In addition, proteome of *L. paracasei* ATCC 334 was retrieved from the NCBI (accession number CP000423.1), and that of *B. longum* ATCC 15707 from Uniprot (Proteome ID: UP000007255).

#### Analysis of Bacteriocins, Antimicrobial Resistance Genes and Virulence Factors

Bacteriocin presence in the diverse genomes was investigated using BAGEL4 webserver (Van Heel et al., 2018). The presence of antimicrobial resistance (AMR) genes was examined in January 2021 through the last version of the Comprehensive Antibiotic Resistance Database (CARD) (Alcock et al., 2020). Additionally and following EFSA criteria (EFSA, 2021), genomic sequences were also compared against ResFinder 4.0 database (Bortolaia et al., 2020) to identify putative acquired antimicrobial resistance genes and/or chromosomal mutations. Potential pathogenicity and the presence of virulence factors were evaluated with the online tools PathogenFinder (Cosentino et al., 2013) and VFanalyzer (Liu et al., 2019), respectively.

#### Analysis of Genes Involved in Biogenic Amines Production

Gene sequences encoding key enzymes involved in biogenic amines metabolism in different lactic acid bacteria were retrieved from the NCBI and investigated by BLAST (Altschul et al., 1990). These included *tdcA* (tyrosine decarboxylase; GenBank accession number JX204286.1), *cadA* (lysine decarboxylase; AE014075.1), *hdcA* (histidine decarboxylase; AJ749838.1), *aguA* (agmatine deiminase; AF446085.5), and *speF* (ornithine decarboxylase; EF633730.1).

#### Analysis of Bile Salt Hydrolase Genes

The *bsh* nucleotide sequences for *Bifidobacterium* (Kim et al., 2004) were retrieved from the NCBI with accession numbers AY506536, AY604516, and AY604517. To determine the presence of Bile Salt Hydrolase (BSH) in *Pediococcus* strain, as no reference *bsh* sequence could be identified for this genus, genomes were annotated using eggNOG-mapper functional annotation tool (Huerta-Cepas et al., 2019) and inspected for the presence of the BSH enzyme choloylglycine hydrolase (EC 3.5.1.24).

#### Analysis of Adhesins and Adhesion Domains

Bacterial proteomes were screened for the pfam adhesion domains listed in Supplementary Table 1 (Vélez et al., 2007; Zhang et al., 2015) using the Conserved Domains Database (CDD) (Marchler-Bauer et al., 2015). Only hits displaying *e*-values below 1E-4 were considered (Xu and Dunbrack, 2012).

### Acute Oral Toxicity Study

Acute oral toxicities of *B. longum* KABP042 and *P. pentosaceus* KABP041 were tested following OECD Guideline for Testing Chemicals No 420 (OECD, 2002). In short, ten 7-week-old female Wistar rats (Envigo, Oxon, United Kingdom) were housed in groups of 5 with drinking water and standard diet *ad libitum* except for the night before the first administration and for the subsequent 3 h. Lyophilized probiotic strains *B. longum* KABP042 and *P. pentosaceus* KABP041 produced by AB-Biotics S.A. (Barcelona, Spain) were re-suspended in PBS 2 h prior administration. A sighting test was performed for each probiotic strain in a single animal at a starting dose of 2,000 mg/kg (equivalent to 2 × 10^11^ CFU/kg). In absence of toxicity, the same animal and an additional group of 4 animals were treated with the same single dose. In total, five rats were treated with *B. longum* KABP042 and 5 animals with *P. pentosaceus* KABP041. Clinical observations were made 30 min, 1, 2, and 4 h after dosing and then daily for 14 days. Morbidity and mortality checks were made twice daily. Individual body weights were recorded on the day of dosing and on days 7 and 14. At the end of the observation period the animals were euthanized by cervical dislocation and subjected to gross necropsy. An estimate of the acute oral median lethal dose (LD_50_) of the treatments was made. Study followed animal welfare requirements of Envigo—Shardlow Ethical policy and United Kingdom’s Animals (Scientific Procedures) Act 1986 Amendment Regulations 2012.

### Pilot Clinical Study

A prospective, observational, multi-site, pilot study was carried out at Azienda Sanitaria Locale Napoli (Naples, Italy) during 2018. Infants enrolled had a diagnosis of FGID (functional constipation and/or infant colic) according to Rome IV criteria for clinical purposes (Benninga et al., 2016). Constipation was diagnosed in infants with two or more of the following symptoms in a month: 2 or fewer defecations per week, excessive stool retention, painful or hard bowel movements, large-diameter stools or presence of a large fecal mass in the rectum. Infant colic was diagnosed in babies up to 5 months old if recurrent and prolonged periods of crying, fusing or irritability occurred without obvious cause and that cannot be prevented or resolved. Other inclusion criteria included: age from 1 to 10 months, ≥ 37 weeks of gestation at birth and ≥ 2500 g of birth weight. Infants with failure to thrive (weight gain < 100 grams/week on average from birth), major medical problems (e.g., immunodeficiencies, developmental problems or genetic abnormalities), severe gastrointestinal diseases, administered with antibiotics for 4 weeks or probiotics for 2 weeks before or during study were excluded.

Sample size was estimated to observe a difference ≥ 1 unit in gastrointestinal symptoms on the Likert scale accepting an alpha risk of 0.05, and a beta risk of 0.2 in a two-sided test. Assuming a standard deviation of 2 and a 10% dropout rate, 40 subjects were needed.

Forty-three patients were screened and 36 who satisfied inclusion and exclusion criteria were enrolled in the study. Infants received a formula containing *B. longum* KABP042 and *P. pentosaceus* KABP041 (1:1) in sunflower oil which was produced by AB-Biotics S.A. A daily dose of 10 drops (2 × 10^9^ CFU of total bacteria) was administered orally for 14 days, preferably with the first meal of the day. Vials were kept at room temperature during the study. The trial was conducted according to the Helsinki Declaration and Good Clinical Practices. Study protocol was approved by the local ethics committee of Azienda Sanitaria Locale Napoli 3 Sud (Italy) and registered as NCT04944628. Signed informed consent was obtained from the caregivers of all participating infants.

The co-primary outcomes of the study were to assess the safety and tolerability of the probiotic product, and the efficacy measured as the difference in total FGIDs severity (summatory of excessive crying score as indicator of colic severity and constipation score) at the end of study relative to baseline. Secondary outcomes included investigating the differences in excessive crying and constipation severity within infant colic and constipation subpopulations, respectively, and change in parental anxiety after the 14 days of treatment.

Demographic characteristics, delivery mode, type of feeding, and past and current medications were obtained from medical records and anamnesis at baseline visit. Excessive crying and constipation severity were individually scored by the pediatricians using 5-point Likert scales (0: no symptoms, to 4: extremely severe symptoms) at baseline and at the end of the treatment. Parents’ anxiety was recorded through the validated Generalized Anxiety Disorder 7-item (GAD-7) scale (Spitzer et al., 2006) by each caregiver before and after treatment period. Any adverse events experienced by the participant were daily recorded by caregivers in the patient’s data-collection diary.

### Statistical Analysis

Statistical analysis was performed using GraphPad Prism Software 5.0 (GraphPad Software Inc., La Jolla, CA, United States). Data are showed as mean and SD. Data from *in vitro* experiments was analyzed by one-way ANOVA followed by Bonferroni *post hoc* test. Clinical data was assessed for normal distribution by Shapiro-Wilk normality tests. Non-parametric continuous variables were compared with the Wilcoxon signed rank test for paired data. Statistical tests were two-sided, at 5% level of significance. The primary efficacy outcome (total FGID score in all recruited subjects) was assessed by Wilcoxon matched-pairs test both on an intent-to-treat (ITT) basis and a per protocol (PP) basis, to ensure the robustness of the findings. For ITT, missing data were imputed using last observation carried forward (LOCF). Secondary outcomes were assessed on a per protocol (PP) basis. Comparisons of individual variables against baseline were assessed by Wilcoxon matched-pairs test, while comparisons between population subgroups was performed by two-way ANOVA followed by Bonferroni *post hoc* test.

## Results

### Safety Evaluation of *Bifidobacterium longum* KABP042 and *Pediococcus pentosaceus* KABP041

The safety of *B. longum* KABP042 and *P. pentosaceus* KABP041 was assessed by combining *in silico, in vitro* and *in vivo* analyses. Genome sequences for both strains were successfully obtained (Supplementary Table 2) and subjected to *in silico* analyses.

VirulenceFinder analysis did not detect true virulence factors for any of the strains. PathogenFinder returned probabilities of being a human pathogen of 0.172 and 0.223 (1 would correspond to 100% probability of being a pathogen) for *P. pentosaceus* KABP041 and *B. longum* KABP042, respectively, with no matches with pathogenic gene families.

Antibiotic susceptibility experiments revealed that *P. pentosaceus* KABP041 was phenotypically resistant to kanamycin, streptomycin, and tetracycline (Table 1). However, genomic analyses using the two maintained databases CARD and ResFinder did not detect any AMR gene, indicating that such resistances are likely driven by intrinsic mechanisms and therefore are not transferable. *B. longum* strain KABP042 displayed phenotypic resistance to erythromycin and clindamycin (Table 1). Genomic inspection revealed that *B. longum* KABP042 harbors the resistance gene *erm(49)*, which confers resistance to both antibiotics and cannot be mobilized (Martínez et al., 2018). Hence, according to EFSA criteria (EFSA, 2018, 2021), *P. pentosaceus* KABP041 and *B. longum* KABP042 do not pose any concern in relation to potential AMR dissemination.

Analysis of culture supernatants of *B. longum* KABP042 and *P. pentosaceus* KABP041 strains grown in the presence of five biogenic amines precursors demonstrated that the strains did not produce such detrimental compounds. In agreement with these results, genes encoding for the key enzymes responsible for the biosynthesis of putrescine (via agmatine and ornithine), histamine, tyramine, and cadaverine were not found in their genomes. None of the studied strains presented hemolytic or gelatinase activities (data not shown). API ZYM experiments revealed that the two strains display different and complementary lipidic, aminoacidic and carbohydrate enzymatic activities (Supplementary Table 3).

To investigate the potential toxicity of the strains *in vivo*, the acute oral toxicity protocol OECD 420 was performed for the two strains using the highest dose of 2,000 mg/kg (equivalent to 2 × 10^11^ CFU/kg). No signs of systemic toxicity were observed during the study and no macroscopic findings were recorded at necropsy in any animal for any treatment. Moreover, all rats showed a normal body weight gain throughout the experiment (Supplementary Table 4), confirming that strains *B. longum* KABP042 and *P. pentosaceus* KABP041 were safe and non-toxic even if consumed at very high doses.

### Both Probiotic Strains Tolerate Simulated Gastrointestinal Conditions

Tolerance of *B. longum* KABP042 and *P. pentosaceus* KABP041 to the gastric conditions was assessed by exposing the strains to a simulated fast-gastric passage (pH 2.3 for 30 min) and a slow postprandial digestion (pH 3 for 90 min). The two studied strains and the well-known probiotic strain *L. rhamnosus* GG showed a loss < 1 log CFU/mL in both challenges (Table 2). As expected, the 3 strains had a lower survival in the more acidic condition. *B. longum* KABP042 showed a slightly lower resistance to pH 3 condition than control *L. rhamnosus* GG (*p* < 0.05) but tolerated better pH 2.3 challenge.

**TABLE 2:** Tolerance to gastric stress and bile salts.

Strain	Gastric solution at pH 2.3			Gastric solution at pH 3			MRS + bile salts 0.3%		
	Count		Loss	Count		Loss	Count		Loss
	t = 0 min	t = 30 min	t = 0 min—t = 30 min	t = 0 min	t = 90 min	t = 0 min—t = 90 min	t = 0 min	t = 90 min	t = 0 min—t = 90 min
*P. pentosaceus* KABP041	6.79 ± 0.22	5.96 ± 0.18	0.84 ± 0.06	6.89 ± 0.56	6.85 ± 0.53	0.05 ± 0.03	6.25 ± 0.14	6.20 ± 0.15	0.05 ± 0.01
*B. longum* KABP042	6.25 ± 0.14	5.67 ± 0.07	0.58 ± 0.07	6.81 ± 0.69	6.53 ± 0.62	0.28 ± 0.06*	6.79 ± 0.22	6.57 ± 0.27	0.22 ± 0.09
*L. rhamnosus* GG	6.98 ± 0.23	6.19 ± 0.21	0.68 ± 0.08	6.92 ± 0.24	7.09 ± 0.19	−0.17 ± 0.16	7.01 ± 0.42	6.85 ± 0.26	0.15 ± 0.24

*p < 0.05.

*Values presented are means and standard deviations of log CFU/mL. Statistical differences between probiotic strains and control L. rhamnosus GG strain were determined by one-way ANOVA followed by Bonferroni’s Multiple Comparison test.*

Furthermore, *B. longum* KABP042 and *P. pentosaceus* KABP041 were found to be highly tolerant to bile salts with a loss < 0.5 log CFU/mL and showing no significant differences with control strain (Table 2). In line with these results, one copy of the *bsh* gene -encoding for a Bile Salt Hydrolase enzyme- was identified in each genome, corroborating that both strains are adapted to the gastrointestinal tract.

### *Bifidobacterium longum* KABP042 and *Pediococcus pentosaceus* KABP041 Adhere to the Intestinal Epithelium

**FIGURE 1:**
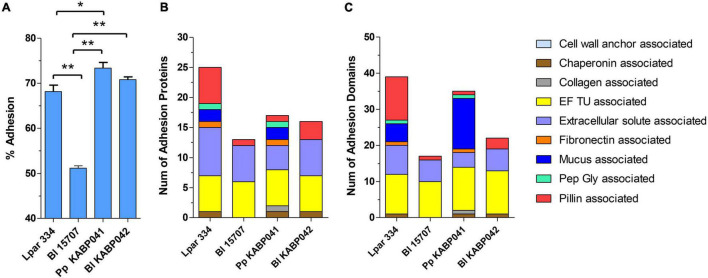
**(A)** Adhesion capacity to Caco-2 cells, **(B)** number of adhesion proteins and **(C)** number of total adhesion domains of *L. paracasei* ATCC 334 (Lpar 334), *B. longum* ATCC 15707 (Bl 15707), *P. pentosaceus* KABP041 (Pp KABP041) and *B. longum* KABP042 (Bl KABP042). Statistical differences were assessed by one-way ANOVA followed by Bonferroni’s Multiple Comparison test. *p < 0.05; **p < 0.001.

Adhesion experiments indicated that both strains efficiently adhere to human intestinal epithelial cells Caco-2. *P. pentosaceus* KABP041 showed 73.4% adherence and *B. longum* KABP042 70.8% (Figure 1A). Both probiotic strains adhered slightly greater than the moderately adherent control strain *L. paracasei* ATCC 334 (*p* < 0.05 for *P. pentosaceus* KABP041) and significantly more (*p* < 0.001 for both strains) than slightly adherent control *B. longum* ATCC 15707 (Sagheddu et al., 2020).

*In silico* analyses revealed that all strains are equipped with an array of predicted adhesion proteins and domains (Figures 1B,C). Genomic analysis results for *B. longum* ATCC 15707, *B. longum* KABP042 and *P. pentosaceus* KABP041 mostly mirrored *in vitro* results, as number of adhesion proteins and domains correlated with their adhesion capacity. Of note, *P. pentosaceus* KABP041 encode for two mucus-binding proteins containing 14 Mub_B2 or MucBP_2 domains, 1 Pep-Gly associated domain, 1 collagen associated domain and 1 fibronectin associated domain, whereas *B. longum* KABP042 encode for a larger number of pilin-associated proteins and domains. These results suggest that both strains may occupy a different niche in the GIT tract.

### *Bifidobacterium longum* KABP042 *and Pediococcus pentosaceus* KABP041 Synergistically Modulate the Expression of Tight Junction Related-Genes

**FIGURE 2:**
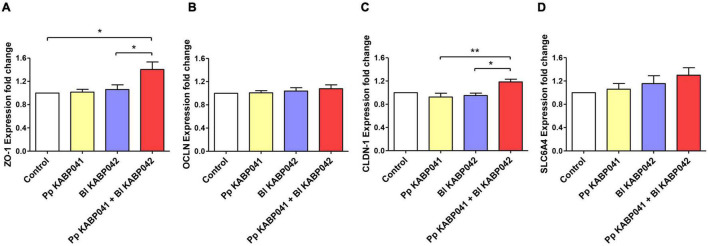
Gene expression of Caco-2 genes **(A)** ZO-1, **(B)** OCLN, **(C)** CLDN-1 and **(D)** SLC6A4 after co-culturing with *P. pentosaceus* KABP041 (Pp KABP041), *B. longum* KABP042 (Bl KABP042) and with a mixture 1:1 of both strains (Pp KABP041 + Bl KABP042). Control condition without bacteria was set at level 1. Statistical differences were assessed by one-way ANOVA followed by Bonferroni’s Multiple Comparison test. *p < 0.05; **p < 0.01.

RT-qPCR experiments on selected Caco-2 genes [i.e., tight junction (TJ) proteins zonula occludens-1, occludin, claudin-1, and the serotonin transporter SERT] were performed to investigate probiotic-host interaction. The presence of *B. longum* KABP042 or *P. pentosaceus* KABP041 did not affect ZO-1 gene expression significantly (Figure 2A). However, exposure of Caco-2 to a mixture (1:1) of both strains resulted in a low but significant upregulation of ZO-1 compared to control (*p* < 0.05) and single strain conditions (*p* < 0.05 vs. *B. longum* strain). OCLN expression showed a similar trend although differences did not reach statistical significance (Figure 2B). Similar to ZO-1, CLDN-1 had a low but still significant increased expression in response to the two-strains combination compared to *B. longum* KABP042 (*p* < 0.05) or *P. pentosaceus* KABP041 (*p* < 0.01) alone (Figure 2C). Although differences were not significant, an interesting trend was observed for the expression of the gene encoding serotonin transporter SERT. SLC6A4 transcript was slightly modulated by the presence of the probiotic strains with the mixture of the two strains having a stronger upregulating effect (Figure 2D). These results indicated that *B. longum* KABP042 and *P. pentosaceus* KABP041 synergistically modulate Caco-2 genes, with significant effects in genes involved in TJ formation.

### Both Probiotic Strains Inhibit the Growth of Gram-Positive and Gram-Negative Pathogens via Production of Organic Acids and Antimicrobials

**FIGURE 3:**
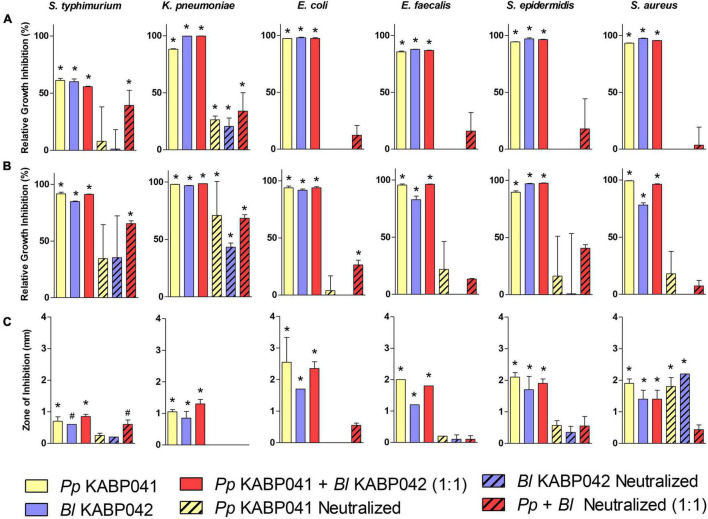
Antagonism activity of *P. pentosaceus* KABP041 (*Pp* KABP041), *B. longum* KABP042 (*Bl* KABP042), and a combination 1:1 of both strains (*Pp* KABP041 + *Bl* KABP042) against diverse Gram-positive and Gram-negative bacterial pathogens. Percentage of growth inhibition (Area Under the Curve) of pathogens cultured in supernatants of **(A)** probiotic mono-cultures, and **(B)** probiotic + pathogen co-cultures relative to pathogen’s growth in control condition. **(C)** Growth inhibition of bacterial pathogens measured in mm determined by the soft agar overlay assay. Free pH and neutralized conditions are indicated. Statistical analyses were performed by the one-way ANOVA with Bonferroni’s Multiple Comparison Test. *p < 0.001; ^#^*p* < 0.05.

Antagonistic activity of supernatants of *B. longum* KABP042 and *P. pentosaceus* KABP041 axenic cultures were investigated with and without neutralization, alone or in combination (1:1) against a panel of bacterial pathogens (Figure 3A). All crude *B. longum* KABP042 and *P. pentosaceus* KABP041 culture supernatants and their combination drastically inhibited the planktonic growth of bacterial pathogens (*p* < 0.0001). Neutralization of individual culture supernatants resulted in the loss of antagonistic activity against *E. coli, E. faecalis, S. epidermidis* and *S. aureus* strains, but still retained inhibitory effect against *K. pneumoniae*, and *S. typhimurium* (*p* < 0.0001). Interestingly, neutralized mixed supernatants (*B. longum* KABP042 plus *P. pentosaceus* KABP041) maintained antagonistic effect against all pathogens, reaching statistical significance against growth of *K. pneumoniae* and *S. typhimurium*. These results indicated that the main antagonistic activity of *B. longum* KABP042 and *P. pentosaceus* KABP041 supernatants is due to the accumulation of organic acids, but also suggested the presence of additional inhibitory compounds that may act synergistically when combined.

To investigate whether the production of antimicrobial compounds is induced by the presence of pathogenic bacteria, co-cultures of *B. longum* KABP042 and *P. pentosaceus* KABP041 with the aforementioned pathogens were performed and their supernatant activity was evaluated (Figure 3B). The results obtained were very similar to those of the mono-culture supernatants, indicating that potential antimicrobials produced by *B. longum* KABP042 and *P. pentosaceus* KABP041 are not induced by the presence of most pathogens investigated. Of note, and in line with mono-culture results, *B. longum* KABP042 and *P. pentosaceus* KABP041 seems to markedly produce antimicrobial compounds against *K. pneumoniae* and *S. typhimurium* when grown in the presence of such pathogens. Such activity appears to be independent of organic acid accumulation, suggesting that production of antimicrobials by *B. longum* KABP042 and *P. pentosaceus* KABP041 is targeted to and induced upon sensing these two particular pathogens.

Finally, the soft agar overlay antagonistic assay was conducted to evaluate the antimicrobial activity of probiotic strains during sessile growth (Figure 3C). As in liquid culture supernatants, both probiotic strains were able to inhibit growth of all pathogens when grown on agar media. Inhibition of pathogens’ growth was mainly mediated by the production of organic acids as well, since neutralization of the soft agar inoculated with the pathogen reduced the zone of inhibition produced by *B. longum* KABP042 and *P. pentosaceus* KABP041 strains. Nonetheless, for *S. aureus, S. epidermidis*, and *S. typhimurium*, the antagonistic activity of both probiotic strains was retained to some extent even under buffered conditions, indicating that antimicrobial compounds are also produced by *B. longum* KABP042 and *P. pentosaceus* KABP041 during sessile growth. Moreover, *B. longum* KABP042 and *P. pentosaceus* KABP041 displayed synergistic antagonism independent of organic acid production against *E. coli* and *S. typhimurium* (*p* < 0.001) when grown in combination on agar media.

Overall, antagonism experiments indicated that the main inhibitory activity of *B. longum* KABP042 and *P. pentosaceus* KABP041 is mediated by the production of organic acids, but neutralization experiments strongly suggested that both strains can produce additional antimicrobial compounds that display an accumulative effect when combined.

In line with this, the biosynthesis of organic acids was confirmed in individual culture supernatants. *B. longum* KABP042 produced 36.1 ± 1.1 mM lactate (100% L-lactate isomer) and 64.9 ± 3.3 mM acetate (ratio lactate 1: acetate 1.8) while *P. pentosaceus* KABP041 accumulated 107 ± 1.7 mM lactate (77% L-lactate) and no acetate. Moreover, the genomes of *B. longum* KABP042 and *P. pentosaceus* KABP041 were screened for the presence of bacteriocins using BAGEL4 tool. While no bacteriocin genes were detected in the genome of *P. pentosaceus* KABP041 through this analysis, the gene encoding for the bacteriocin Lantibiotic B (Bisin) was identified in the genome of *B. longum* KABP042 and further BLAST analyses revealed the presence of the accessory genes *lanR2, lanK, lanA, lanR1*, and *lanI* (data not shown). These results evidence that, beside accumulation of organic acids, the probiotic strains could at least produce the bacteriocin Lantibiotic B, which displays broad antimicrobial spectrum (Lee et al., 2011). To confirm the proteinaceous nature of the antimicrobials, neutralized mono-culture supernatants were treated with proteases and retested against the indicator strain *K. pneumoniae* DSM 11678. The results strongly suggested that both probiotic strains produce antimicrobials of proteinaceous nature as treated supernatants significantly lost inhibitory activity (Supplementary Figure 1).

### Safety and Efficacy of *Bifidobacterium longum* KABP042 and *Pediococcus pentosaceus* KABP041 in Reduction of Functional Gastrointestinal Disorder Severity in Infants

Thirty-six infants aged between 1 and 40 weeks, meeting inclusion and exclusion criteria, were enrolled in the pilot study. Main demographic and clinical features at baseline are shown in Table 3. No infant changed feeding type during study period. Two participants discontinued the study, one of them because of experiencing intestinal spams and facial redness. Withdrawal cause of the other participant was unknown. These were the only adverse events reported which were mild in intensity and rapidly mitigated with no medication needed. No serious adverse events were observed, indicating the safety and tolerability of the probiotic mixture.

**TABLE 3:** Demographic and clinical characteristics of recruited subjects (*n* = 36) at enrollment and number of patients using concomitant medication during trial period.

Age (weeks)	12.92 ± 8.67
Weeks of gestation at birth	38.57 ± 2.05
Weight (kg)	3.16 ± 0.48
Gender (Females/Males)	23 (63.88%)/13 (36.12%)
Feeding mode (breastfeeding/mixed/formula)	13 (36.11%)/9 (25%)/13 (36.11%)
Delivery mode (vaginal/C-section)	17 (47%)/19 (53%)
Previous medication (Yes)	12 (33.33%)
Concomitant medication (Yes)	11 (30.55%)
FGID incidence (infant colic/constipation)	30 (83.33%)/23 (64%)
Total FGID severity score	3.65 ± 1.48
Excessive crying score	2.13 ± 0.83
Constipation severity score	1.84 ± 1.09

*Data are presented as mean ± SD or absolute number and percentages. Feeding mode data was missed for one patient. Previous and concomitant medication included simethicone, hydrolyzed formula, herbal extracts and/or laxatives.*

**FIGURE 4:**
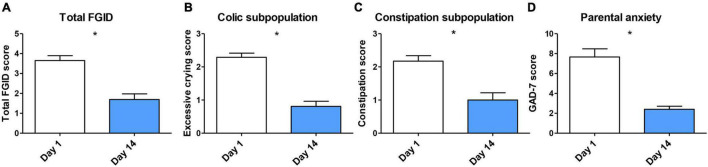
(A) Overall FGID amelioration after probiotic treatment analyzed by ITT (*n* = 36–32), missing data was filled with LOCF. PP analysis of (B) excessive crying reduction in colic subpopulation (*n* = 26); (C) constipation reduction in constipation subpopulation (*n* = 23); and (D) parental anxiety reduction (*n* = 50) after treatment. Data are presented as means ± SD. Statistical differences were analyzed by Wilcoxon matched pairs test. *p < 0.0001.

ITT analysis revealed statistically significant differences in total FGID severity score between baseline (3.65 ± 1.48) and end of the treatment (1.69 ± 1.70, *p* < 0.0001; Figure 4A). PP analysis confirmed these significant differences (*n* = 32, 3.58 ± 1.49 vs. 1.38 ± 1.45, *p* < 0.0001). Then, we investigated changes in excessive crying and constipation scores in correspondingly colicky and constipated patients’ subpopulations by PP analysis. A significant reduction of excessive crying score (2.29 ± 0.64 at baseline vs. 0.81 ± 0.80 at day 14, *p* < 0.0001) and constipation severity score (2.18 ± 0.79 at baseline vs. 1.00 ± 1.00 at day 14, *p* < 0.0001) was observed (Figures 4B,C). These outcomes indicate that probiotic treatment may reduce FGID severity in infants suffering colic and constipation symptoms.

There is evidence of the role of parental anxiety in the incidence of FGIDs such as infant colic (Thomas et al., 2016). We assessed parent’s anxiety before and after infant’s probiotic administration. Parents presented a reduced anxiety state at the end of the study. GAD-7 score was significantly lower at day 14 in comparison with baseline (7.66 ± 5.77 vs. 2.40 ± 2.21, *p* < 0.0001; Figure 4D).

We further evaluated whether feeding type, delivery mode and administration of previous or concomitant medications affected FGID amelioration under probiotic therapy. We analyzed changes in total FGID symptoms in subgroups of breastfed, formula-fed, mixed-fed, vaginally delivered, C-section delivered, previously medicated and unmedicated, concomitant treated and untreated babies. Significance of the primary endpoint (total FGID severity) was retained in all the subgroups studied (Supplementary Table 5). Analysis of score changes in subgroups within colic and constipation subpopulations also found significant reduction of excessive crying score and constipation severity score after probiotic treatment for all variables analyzed (*p* < 0.0001 for time and *p* > 0.05 for interaction effects).

## Discussion

Insights about gut barrier disfunction and microbiome perturbations in the pathophysiology of FGIDs support a strong rationale to use specific probiotic strains as potential therapies due to their interactions with host intestinal epithelium and microbes among other roles (Pärtty et al., 2018). A number of strains have been proposed for the management of pediatric FGIDs (Wojtyniak and Szajewska, 2017; Sung et al., 2018; Nocerino et al., 2020; Savino et al., 2020). However, no specific probiotic strains have gained enough clinical evidence to be recommended by experts except for *Limosilactobacillus reuteri* DSM 17938 but only in breastfed babies subgroup (Tabbers et al., 2014; Guarner et al., 2017; Martinelli et al., 2020). In addition to the limited clinical support, strains are not always selected based on beneficial mechanisms to counteract pediatric FGIDs pathophysiology but generally tested in different adult and infant conditions. As stated by FAO/WHO (Morelli and Capurso, 2012) and ISAAP (Hill et al., 2014), several probiotic benefits are strain-specific and thus the mechanistic basis should be studied. In this scenario, a demand of evidence-based probiotic formulations with functional-characterized specific strains for pediatric disorders is increasing.

In this study, we investigated the probiotic properties and the functionality of the two strains *B. longum* KABP042 and *P. pentosaceus* KABP041 through *in vitro* tests, genomic analysis, and *in vivo* assays, including an exploratory, observational trial to test the safety, tolerability and efficacy of the probiotic combination in infants diagnosed with FGIDs involving infant colic and functional constipation.

In accordance to the Qualified Presumption of Safety (QPS) status of *B. longum* and *P. pentosaceus* species (EFSA, 2020), the safety assessment of the two probiotic strains concluded no virulence determinants were present in the genomes, and no strain displayed hemolytic or gelatinase activity nor produced biogenic amines, fulfilling safety prerequisites (Sanders et al., 2010). Although *P. pentosaceus* KABP041 displayed MIC levels above defined cut-off values to kanamycin, streptomycin and tetracycline (Table 1), no genetic basis was identified by using the two maintained antimicrobial databases CARD and ResFinder as required by EFSA (2021). High MIC values to these antimicrobials without correlated gene were also observed in pediococci strains by other researchers indicating resistance is due to unknown natural mechanisms (Danielsen et al., 2007; Klare et al., 2007; Lüdin et al., 2018; Diguţă et al., 2020). As suggested by these authors, our results also indicate current recommended EFSA cut-off values for *Pediococcus* sp. are below natural MIC levels for some antimicrobials and those breakpoints should be revised. *B. longum* KABP042 showed atypical resistance to erythromycin and clindamycin and the resistance gene *erm(49)* was identified in its genome. Recent research has shown that the gene *erm(49)* encodes for a rRNA methylase that confers resistance to erythromycin and clindamycin and appears to be restricted to very few strains of *B. longum* and *B. breve*. Importantly, conjugation experiments revealed that this gene is not transferable, thus discarding the risk of potential dissemination (Martínez et al., 2018). Together, these results fulfill safety criteria for the use of *B. longum* KABP042 and *P. pentosaceus* KABP041 as probiotics (Sanders et al., 2010; Morelli and Capurso, 2012; EFSA, 2018, 2021). In addition, the safety status of the studied probiotics was validated in rats fed with a very high acute dose of each strain and later in a pilot study involving 36 infants.

Through the *in vitro* characterization, we confirmed that *B. longum* KABP042 and *P. pentosaceus* KABP041 efficiently tolerate gastric and bile salts stress and adhere to human intestinal epithelial cells, which are essential requirements for probiotic selection as recommended by guidelines (Morelli and Capurso, 2012). Interestingly, the two strains seem to exploit different adhesion mechanisms to attach to Caco-2 intestinal cells. While adhesins in *P. pentosaceus* KABP041 appear to be more varied and dominated by mucus-binding proteins, the genome of *B. longum* KABP042 harbors a larger number of pili (Figure 1). It is remarkable that strain *P. pentosaceus* KABP041, which presents the shorter genome (1.7 Mb), contains larger number and greater diversity of adhesion determinants compared to *B. longum* strains (∼2.3 Mb). The elevated number of adhesion proteins and domains identified in control strain *L. paracasei* ATCC 334 may be in part attributed to its large genome size (> 3 Mb) although it did not translate to a better adherence than the *Pediococcus* strain. These results may explain the elevated adhesion capacity of *P. pentosaceus* KABP041 but also suggest that this strain is more versatile in terms of binding to different substrates in the gastrointestinal tract. Indeed, the efficient adhesion of pediococci to the non-mucus producing HT29 cells have been reported, although results indicated a preferent adhesion to the intestinal mucus-producing cells HT29-MTX (Turpin et al., 2012), highlighting the biological importance of mucus-binding domains and suggesting KABP041 adhesion could be greater in *in vivo* models. There is also evidence that bifidobacterial pili play a central role in the colonization of the gut and attachment to intestinal cells (Westermann et al., 2016). In fact, pili structures serve as virulence factors of many bacterial pathogens for the initial attachment to intestinal tissues (Telford et al., 2006; Proft and Baker, 2009). Therefore, pili production by bifidobacteria may have a dual role: colonization of the intestine and exclusion of bacterial pathogens, as reported for other probiotics (Tytgat et al., 2016; Ligthart et al., 2020).

Probiotic adhesion to intestinal cells facilitates a close dialog with the host. We investigated this by measuring the expression of markers of barrier function in Caco-2 cells including TJ proteins zonula occludens-1, occludin and claudin-1 which are crucial for the maintenance of barrier integrity (Chelakkot et al., 2018). In addition, serotonin transporter SERT expression was quantified. This protein is involved in serotonin reuptake and therefore in gut motility and sensibility and its downregulation is implicated in the pathophysiology of FGIDs such as irritable bowel syndrome (Jin et al., 2016). We illustrated *B. longum* KABP042 and *P. pentosaceus* KABP041 modulate the gene expression of intestinal cells (Figure 2), genes involved in TJ formation being particularly induced by the presence of the probiotic combination. Interestingly, a trend in serotonin transporter SERT expression induction was observed. Further experiments are needed to confirm the potential role of KABP042 and KABP041 combination in favoring serotonin uptake. Although the upregulation of TJ protein genes by probiotics is described elsewhere for single (Anderson et al., 2010) and combined strains (Rijcken, 2009), the potential synergic effects are less studied. In this regard, Shiou et al. (2013) showed combined lactobacilli and bifidobacteria strains had synergistic effects over TJ expression. However, the mechanisms behind are unknown. The diversity of adhesion proteins encoded by *B. longum* KABP042 and *P. pentosaceus* KABP041 might be related with the synergistic modulation of Caco-2 genes.

Abnormal enterobacterial levels are linked to gut dysbiosis in FGIDs such as infant colic (Savino et al., 2017; Rhoads et al., 2018). We have shown that both *B. longum* KABP042 and *P. pentosaceus* KABP041 can antagonize enteropathogens (Figure 3). In addition, we have demonstrated the probiotic strains also inhibit the growth of Gram-positive microbes causative of pediatric infections (Cortese et al., 2016), emphasizing the potential benefits of the studied probiotics in the first stages of life. Such inhibitory activities seem mainly mediated by the production of the organic acids lactate and/or acetate, but neutralization and proteolysis of culture supernatants strongly suggested that both probiotic strains can produce bacteriocins. Although further research is required to confirm bacteriocin production and identify the precise molecule(s), the proteolysis of culture supernatants and *in silico* data suggest that *B. longum* KABP042 can at least produce the Lantibiotic B.

In accordance with our observations, Lee et al. (2011) studied this bacteriocin in *B. longum* strain DJO10A revealing a broad inhibition range. However, DJO10A only expressed Lantibiotic B during growth on agar media while our results suggest *B. longum* KABP042 can produce antimicrobials both in agar and liquid cultures. Although less studied than lactobacilli, the production of bacteriocins by pediococci has been described (Papagianni and Anastasiadou, 2009). Our experiments also suggest *P. pentosaceus* strain can produce acid-independent antimicrobials. However, this effect does not correlate with a genetic basis maybe due to few *Pediococcus* sp. genomes are included in BAGEL4 database. Interestingly, the combination of *B. longum* KABP042 and *P. pentosaceus* KABP041 produced higher inhibition than strains alone in some pathogens, especially in neutralized conditions. This outcome suggests a potential antimicrobial-induction and/or synergistic effect between probiotic strains that will need further investigation. Similarly, Gutiérrez-Cortés et al. (2018) indicated an enhanced bacteriocin production by *P. pentosaceus* strain 147 in presence of a *Lactiplantibacillus plantarum* inducer strain.

Our observational pilot study demonstrated that the oral intake of *B. longum* KABP042 and *P. pentosaceus* KABP041 mixture for 14 days was well-tolerated by infants diagnosed with FGIDs (infant colic and/or functional constipation). Moreover, infant FGID severity and parental anxiety were significantly reduced according to physicians and caregivers rating, respectively (Figure 4). Our pilot study, however, present some limitations including the absence of placebo group, but also de lack of daily information on exact crying time and number of bowel movements. Moreover, infants were diagnosed according to Rome IV criteria for clinical practice although not for investigation (Benninga et al., 2016). Despite described limitations, we found additional interesting outcomes by investigating FGID severity reduction in different subgroups within the studied population. Treated infants improved FGID symptoms regardless they were born vaginally, by C-section, fed with human milk, formula, or both, and treated with other medications before or during probiotic therapy. In contrast, *L. reuteri* DSM 17938 efficacy in infant colic is limited to breastfed babies. Reasons are unknown, but were hypothesized to be related to microbiota composition (Sung et al., 2018). Of note, a recently published, randomized, double-blind, placebo-controlled study including a larger population of mainly breastfed colicky infants validated the efficacy of the strains *B. longum* KABP042 and *P. pentosaceus* KABP041 in reducing crying time and number of crying episodes (Chen et al., 2021). Still, the effect of patient’ characteristics and the efficacy of the probiotic formula in infants with functional constipation was not covered in the last study. Our work deeply describes the probiotic properties of strains *B. longum* KABP042 and *P. pentosaceus* KABP041 and provides mechanistic basis underlying the efficacy of the probiotic formula to treat infant FGID (Santas et al., 2015; Chen et al., 2021).

## Conclusion

In conclusion, this study of characterization of *B. longum* KABP042 and *P. pentosaceus* KABP041 confirms that the strains fulfill probiotic requisites and show functionality over intestinal barrier integrity and pathogen inhibition. In addition, the outcome of the observational, single-arm, pilot trial confirms the safety of the probiotic formula and set the basis for future larger, randomized, double blinded, placebo-controlled clinical trials in infants with diverse FGIDs.

## Data Availability Statement

The datasets presented in this study can be found in online repositories. The names of the repository/repositories and accession number(s) can be found below: https://www.ncbi.nlm.nih.gov/genbank/, JAHTMM000000000; https://www.ncbi.nlm.nih.gov/genbank/, JAHTKJ000000000.

## Ethics Statement

The studies involving human participants were reviewed and approved by the Azienda Sanitaria Locale Napoli 3 Sud Servizio Coordinamento Comitato Etico Campania Sud. Written informed consent to participate in this study was provided by the participants’ legal guardian/next of kin. The animal study was reviewed and approved by Envigo—Shardlow Ethical policy and United Kingdom’s Animals (Scientific Procedures) Act 1986 Amendment Regulations 2012.

## Author Contributions

JE-M and MP conceived the idea. EA and TA conducted the *in vitro* experiments. PH performed the *in silico* analysis. MS enrolled and performed patient procedures. EA, PH, MA, and MP analyzed the results. MP and PH wrote the manuscript. All authors reviewed the manuscript.

## Conflict of Interest

EA, PH, TA, MA, MP and JE-M were employees of AB-Biotics S.A. (part of KANEKA Corporation). The isolates described in this study are the subject of a pending patent application co-authored by MP and JE-M. This study received funding from AB-Biotics S.A. The funder had the following involvement with the study: design, data collection, analysis, decision to publish and preparation of the manuscript. The remaining author declares that the research was conducted in the absence of any commercial or financial relationships that could be construed as a potential conflict of interest.

## Publisher’s Note

All claims expressed in this article are solely those of the authors and do not necessarily represent those of their affiliated organizations, or those of the publisher, the editors and the reviewers. Any product that may be evaluated in this article, or claim that may be made by its manufacturer, is not guaranteed or endorsed by the publisher.
