# Supplementary material for: Probiotic Properties of Bifidobacterium longum KABP042 and Pediococcus pentosaceus KABP041 Show Potential to Counteract Functional Gastrointestinal Disorders in an Observational Pilot Trial in Infants
Source: Front Microbiol. 2022 Jan 12;12:741391. doi: 10.3389/fmicb.2021.741391 (PMC8790238; doi:10.3389/fmicb.2021.741391)
Supplement: Supplementary file 1 [file Data_Sheet_1.docx]

Supplementary Material

**Table S1.** Adhesion domains investigated in this study.

| **pfam ID** | **Domain** | **Short name** | **Classification** |
| --- | --- | --- | --- |
| cl37551 | Collagen_bind superfamily | - | Collagen associated |
| pfam03143 | Elongation factor Tu C-terminal domain 3 | GTP_EFTU_D3 | EF-Tu associated |
| pfam03144 | Elongation factor Tu domain 2 | GTP_EFTU_D2 |  |
| pfam00009 | Elongation factor Tu GTP binding domain | GTP_EFTU |  |
| pfam05833 | fibronectin binding domain | FbpA | Fibronectin associated |
| pfam00039 | Fibronectin Binding Domain | fn1 |  |
| pfam05790 | Immunoglobulin C2-set domain | C2-set | Immunoglobulin associated |
| pfam00047 | Immunoglobulin Binding Domain | Ig |  |
| pfam13519 | von Willebrand factor type A domain | VWA_2 | Pilin associated |
| pfam16555 | Gram-positive pilin subunit D1, N-terminal domain | GramPos_pilinD2 |  |
| pfam17802 | Prealbumin-like fold domain | SpaA |  |
| pfam00114 | Pilin | Pil |  |
| pfam00118 | TCP-1/cpn60 chaperonin family domain | Cpn60_TCP1 | Chaperonin associated |
| pfam00139 | Legume lectin domain | Lectin_legB | Lectin associated |
| pfam00497 | Bacterial extracellular solute-binding domain | SBP_bac_3 | Extracellular solute associated |
| pfam00746 | cell wall anchor motif | LPxTG | Cell wall anchor associated |
| pfam01391 | Collagen triple helix repeat (20 copies) | Collagen | Collagen associated |
| pfam01471 | peptidoglycan binding domain | PG_binding_1 | PepGly associated |
| pfam03217 | SLAP Bacterial S-layer domain | SLAP | S-layer associated |
| pfam05670 | DUF814 Domain | DUF814 | Domain of unknown funtion |
| pfam17965 | Mub B1 Domain | Mub_B1 | Mucus associated |
| pfam17966 | Mub B2 Domain | Mub_B2 |  |
| pfam06458 | Mucin-Binding Protein | MucBP |  |
| pfam07979 | Intimin Binding Domain | Intimin_C | Intimin associated |

**Table S2.** General genomic characteristics of *B. longum* KABP042 and *P. pentosaceus* KABP041.

| **Species** | **Strain** | **Size (bp)** | **N50** | **Contigs** | **GC [%]** | **CDS** | **Proteins** | **rRNA** | **tRNA** | **Accession Num** |
| --- | --- | --- | --- | --- | --- | --- | --- | --- | --- | --- |
| *P. pentosaceus* | KABP041 | 1,706,512 | 288,659 | 12 | 37.3 | 1,652 | 1,598 | 3 | 48 | JAHTMM000000000 |
| *B. longum* | KABP042 | 2,342,780 | 199,715 | 48 | 59.6 | 1,927 | 1,861 | 6 | 55 | JAHTKJ000000000 |

**Table S3.** API-ZYM enzymatic activities of studied strains. Results were validated in 3 independent replicates.

| **Enzyme** | ***P. pentosaceus* KABP041** | ***B. longum***  **KABP042** |
| --- | --- | --- |
| Alkaline phosphatase | **-** | **-** |
| Esterase (C4) | **-** | **+** |
| Esterase Lipase (C8) | **-** | **+** |
| Lipase (C14) | **-** | **-** |
| Leucine arylamidase | **+** | **+** |
| Valine arylamidase | **+** | **-** |
| Cystine arylamidase | low | **-** |
| Trypsin | low | **-** |
| α-Chymotrypsin | **-** | **-** |
| Acid phosphatase | **+** | **+** |
| Naphthol AS-BI-phosphydrolase | **+** | **+** |
| α-galactosidase | **-** | low |
| β-galactosidase | **-** | **+** |
| β-glucoronidase | **-** | **-** |
| α-glucosidase | **-** | low |
| β-glucosidase | **+** | **-** |
| N-acetyl-β-glucosaminidase | **+** | low |
| α-mannosidase | low | **-** |
| α-fucosidase | **-** | **-** |

**Table S4.** Monitored body weights (g) of rats used in the acute oral toxicity studies. Mean and standard deviations of data from 5 animals per treatment.

| **Treatment** | **Body weight (g)** | | |
| --- | --- | --- | --- |
|  | Day 0 | Day 7 | Day 14 |
| *P. pentosaceus* KABP041 | 158.3 ± 4.3 | 189.8 ± 9.1 | 197.7 ± 11.6 |
| *B. longum* KABP042 | 161.2 ± 6.5 | 179.0 ± 6.8 | 194.4 ± 9.9 |

**Table S5.** Post-hoc analysis on total FGID score, excessive crying score and constipation score reduction across study subgroups. Data are presented as mean ± SD. Excessive crying and constipation scores were analyzed within colicky and constipation subpopulations, respectively. Group sizes are indicated. Feeding mode information was missed for one patient. Statistical differences were assayed by two-way ANOVA test adjusted by Bonferroni test. For all variables, p-value<0.0001 for time effect and p-value>0.05 for interaction effect were found. P-values of subgroup differences effect and adjusted p-values of time effect in each subgroup are indicated. na, not applicable.

| **Subgroup** | **Total FGID score (n=32)** | | | | **Excessive crying score (n=26)** | | | | **Constipation score (n=23)** | | | |
| --- | --- | --- | --- | --- | --- | --- | --- | --- | --- | --- | --- | --- |
|  | n | Day 1 | Day 14 | p-value | n | Day 1 | Day 14 | p-value | n | Day 1 | Day 14 | p-value |
| **Feeding mode** |  |  |  |  |  |  |  |  |  |  |  |  |
| Subgroup effect |  | na | na | >0.05 |  | na | na | >0.05 |  | na | na | >0.05 |
| Time effect by subgroup: |  |  |  |  |  |  |  |  |  |  |  |  |
| Breastfeeding | 11 | 3.82±1.5 | 1.09±1.4 | <0.001 | 9 | 2.44±0.5 | 0.44±0.5 | <0.001 | 9 | 2.22±0.7 | 0.89±1.1 | <0.001 |
| Mixed | 7 | 4.00±2.0 | 1.71±2.1 | <0.001 | 5 | 2.20±0.8 | 1.00±1.2 | <0.01 | 5 | 2.40±1.1 | 1.4±1.5 | <0.05 |
| Formula | 13 | 3.08±1.2 | 1.23±0.9 | <0.001 | 11 | 2.23±0.7 | 0.91±0.7 | <0.001 | 8 | 1.94±0.8 | 0.75±0.7 | <0.01 |
| **Delivery mode** |  |  |  |  |  |  |  |  |  |  |  |  |
| Subgroup effect |  | na | na | <0.05 |  | na | na | >0.05 |  | na | na | <0.05 |
| Time effect by subgroup: |  |  |  |  |  |  |  |  |  |  |  |  |
| Vaginal | 14 | 4.25±1.6 | 1.93±1.8 | <0.001 | 13 | 2.31±0.8 | 1.00±0.7 | <0.001 | 10 | 2.65±0.7 | 1.40±1.3 | <0.001 |
| C-section | 18 | 3.06±1.2 | 0.94±0.9 | <0.001 | 13 | 2.27±0.5 | 0.62±0.5 | <0.001 | 13 | 1.810.7 | 0.690.8 | <0.001 |
| **Previous medication** |  |  |  |  |  |  |  |  |  |  |  |  |
| Subgroup effect |  | na | na | >0.05 |  | na | na | >0.05 |  | na | na |  |
| Time effect by subgroup: |  |  |  |  |  |  |  |  |  |  |  |  |
| Yes | 10 | 3.30±1.3 | 1.00±0.8 | <0.001 | 10 | 2.30±0.7 | 0.80±0.6 | <0.001 | 5 | 2.00±1.0 | 0.40±0.5 | <0.001 |
| No | 22 | 3.70±1.6 | 1.55±1.7 | <0.001 | 16 | 2.28±0.6 | 0.81±0.9 | <0.001 | 18 | 2.22±0.8 | 1.17±1.1 | <0.001 |
| **Concomitant medication** |  |  |  |  |  |  |  |  |  |  |  |  |
| Subgroup effect |  | na | na | >0.05 |  | na | na | >0.05 |  | na | na | <0.05 |
| Time effect by subgroup: |  |  |  |  |  |  |  |  |  |  |  |  |
| Yes | 10 | 3.60±1.9 | 1.40±2.0 | <0.001 | 9 | 2.33±0.7 | 0.78±1.1 | <0.001 | 4 | 3.00±0.8 | 1.75±1.5 | <0.05 |
| No | 22 | 3.57±1.3 | 1.36±1.2 | <0.001 | 17 | 2.26±0.6 | 0.82±0.6 | <0.001 | 19 | 2.00±0.7 | 0.84±0.9 | <0.001 |


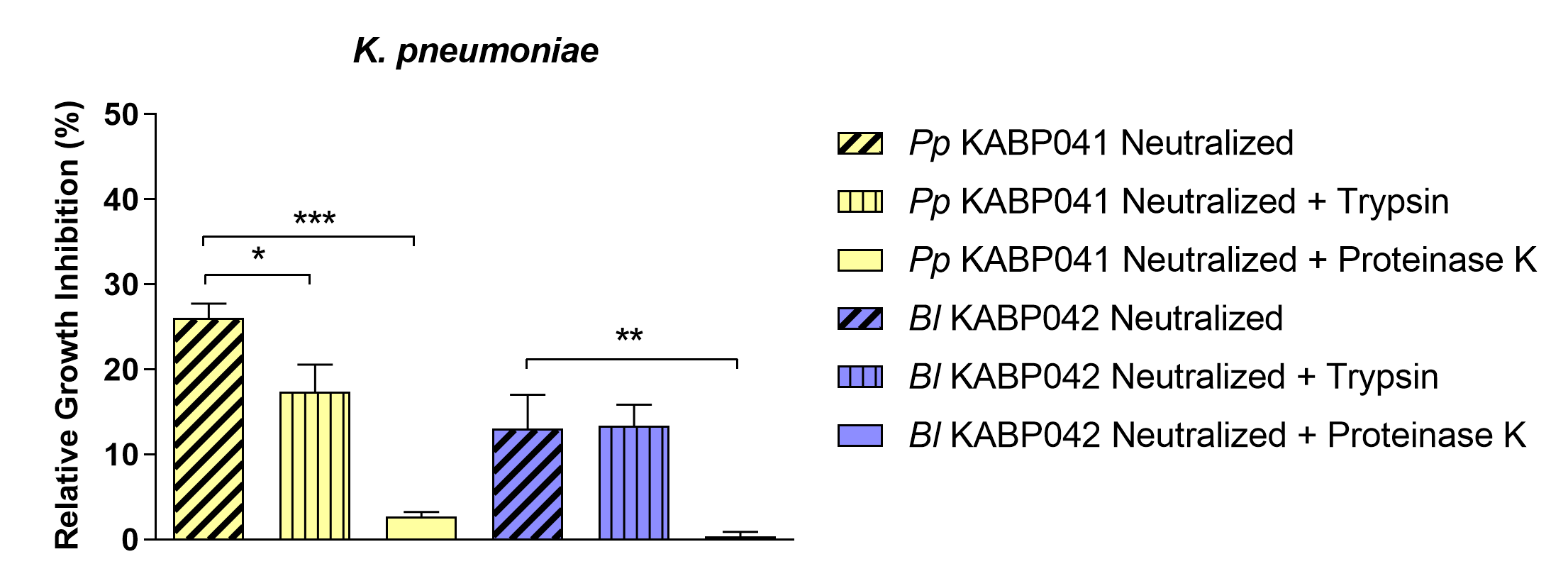


**Figure S1.** Percentage of growth inhibition (Area Under the Curve) of *K. pneumoniae* cultured in supernatants of *P. pentosaceus* KABP041 (*Pp* KABP041) and *B. longum* KABP042 (*Bl* KABP042) after neutralization (control) and after neutralization and enzymatic treatment with trypsin or proteinase K. Statistical analyses were performed by the one-way ANOVA with Bonferroni's Multiple Comparison Test.***, p-value <0.0001**, p-value<0.001; *, p-value<0.05.
